# [Corrigendum] Effects of 17-allylamino-17-demethoxygeldanamycin on the induction of apoptosis and cell cycle arrest in HCT-116 cells

**DOI:** 10.3892/ol.2026.15507

**Published:** 2026-03-03

**Authors:** Xuerong Zhao, Jianping Wang, Lijun Xiao, Qian Xu, Enhong Zhao, Xin Zheng, Huachuan Zheng, Shuang Zhao, Shi Ding

Oncol Lett 14: 2177–2185, 2017; DOI: 10.3892/ol.2017.6442

Following the publication of the above article, an interested reader drew to the authors' attention that, concerning the mRNA electrophoretic blots shown in Fig. 5A on p. 2182, the middle and right-hand blots for Cyclin D1 were similar to the left-hand and middle blots for Caspase 9; in addition, the left-hand Cyclin D1 blot was similar to the right-hand blot for GAPDH in the same figure part.

In response to the reader's enquiry, the authors have submitted a revised version of Fig. 5, now showing alternative data for the Cyclin D1 and GAPDH experiments in Fig. 5A, as presented below. The authors can confirm that any errors associated with this figure did not have a significant impact on either the results or the conclusions reported in this study, and all the authors agree with the publication of this Corrigendum. The authors are grateful to the Editor of *Oncology Letters* for allowing them the opportunity to publish this Corrigendum; furthermore, they apologize to the readership of the Journal for any inconvenience caused.

## Figures and Tables

**Figure 5.**
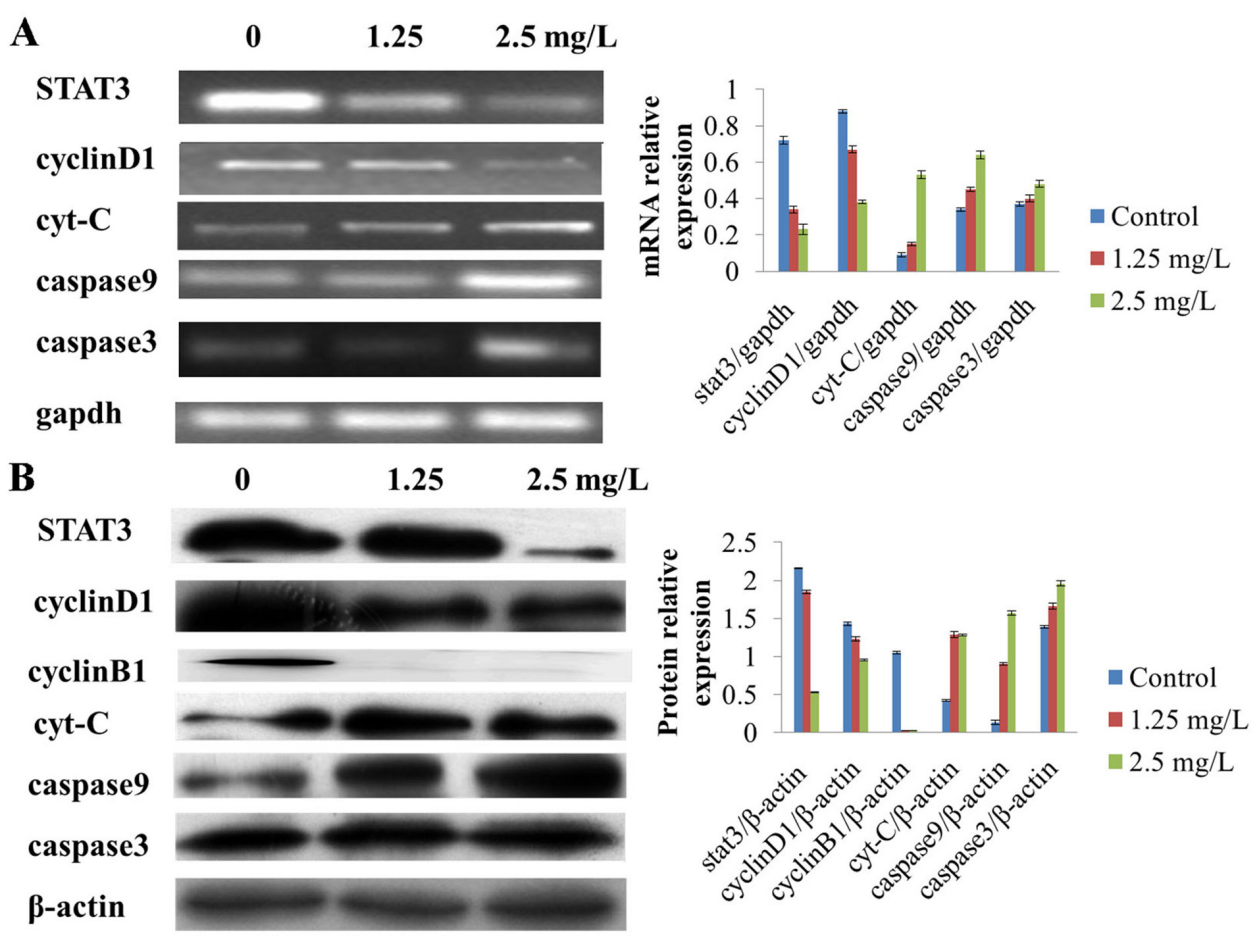
Effect of 17-AAG on the expression levels of various cellular factors. (A) Following treatment with 17-AAG, cyt-c, caspase 9 and caspase 3 mRNA expression levels were increased in HCT-116 cells in a concentration-dependent manner compared with the control. By contrast, STAT3 and cyclin D1 mRNA expression levels were decreased in a concentration-dependent manner, compared with the control. (B) Following 17-AAG-treatment, cyt-c, caspase 9 and caspase 3 protein expression was significantly increased in a concentration-dependent manner, compared with the control, whereas stat3, cyclin D1 and cyclin B1 expression was significantly decreased in a concentration-dependent manner, compared with the control. *P<0.05 compared with the control. 17-AAG, 17-allylamino-17-demethoxygeldanamycin; mRNA, messenger RNA; cyt-c, cytochrome *c*; STAT3, signal transducer and activator of transcription 3.

